# Rapid and safe one-step extraction method for the identification of *Brucella* strains at genus and species level by MALDI-TOF mass spectrometry

**DOI:** 10.1371/journal.pone.0197864

**Published:** 2018-06-05

**Authors:** Michela Sali, Flavio De Maio, Michela Tarantino, Giuliano Garofolo, Manuela Tittarelli, Lorena Sacchini, Katiuscia Zilli, Paolo Pasquali, Paola Petrucci, Cinzia Marianelli, Massimiliano Francia, Maurizio Sanguinetti, Rosanna Adone

**Affiliations:** 1 Insitute of Microbiology, Fondazione Policlinico A. Gemelli - IRCCS - Università Cattolica del Sacro Cuore, Rome, Italy; 2 Department of Veterinary Public Health and Food Safety, Istituto Superiore di Sanità, Rome, Italy; 3 National and OIE Reference Laboratory for Brucellosis, Istituto Zooprofilattico Sperimentale dell’Abruzzo e del Molise “G. Caporale”, Teramo, Italy; Universidad Nacional, COSTA RICA

## Abstract

Brucellosis is essentially a disease of domesticated livestock; however, humans can also be infected via the consumption of contaminated meat or dairy products, underlying the need for rapid and accurate identification methods. Procedures for microbiological identification and typing of *Brucella* spp. are expensive, time-consuming, and must be conducted in biohazard containment facilities to minimize operator risk. The development of a matrix-assisted laser desorption/ionization time-of-flight mass spectrometry (MALDI-TOF-MS)-based assay has reduced the processing time while maintaining performance standards. In this study, to improve the identification accuracy and suitability of the MALDI-TOF-based assay for routine diagnosis, we developed a new protein extraction protocol and generated a custom reference database containing *Brucella* strains representative of the most widespread species. The reference library was then challenged with blind-coded field samples isolated from infected animals. The results indicated that the database could be used to correctly identify 99.5% and 97% of *Brucella* strains at the genus and species level, respectively, indicating that the performance of the assay was not affected by the different culture conditions used for microbial isolation. Moreover, the inactivated samples were stored and shipped to reference laboratories with no ill effect on protein stability, thus confirming the reliability of our method for routine diagnosis. Finally, we evaluated the epidemiological value of the protocol by comparing the clustering analysis results of *Brucella melitensis* strains obtained via multiple locus variable-number tandem repeat analysis or MALDI-TOF MS. The results showed that the MALDI-TOF assay could not decipher the true phylogenetic tree, suggesting that the protein profile did not correspond with the genetic evolution of *Brucella*.

## Introduction

Brucellosis is still considered the most important global zoonosis. In large parts of the world, including the Mediterranean Basin, North Africa, Mexico, and Central and South America, it remains an important public health problem, and results in severe economic losses for livestock industries [1]. In addition to classical species *Brucella melitensis*, *B*. *abortus*, *B*. *suis*, *B*. *canis*, *B*. *ovis*, and *B*. *neotomae*, several other species have recently been assigned to the genus *Brucella*: *B*. *pinnipedialis* and *B*. *ceti*, isolated from marine mammals [2,3]; *B*. *inopinata*, isolated from a breast implant infection [4]; *B*. *microti*, isolated from wild rodents [5,6], and *B*. *papionis*, isolated from baboons [7], indicating that the host range of this genus is increasing. Moreover, many atypical strains that were originally misidentified using conventional phenotyping approaches have now been identified as *Brucella* using molecular methods [6,8].

*B*. *melitensis*, *B*. *abortus*, *B*. *suis*, and *B*. *ovis*, in order of virulence, can infect humans via the consumption of contaminated dairy products, by direct contact with infected animals, or by inhalation of infected aerosols [1,9]. *B*. *neotomae* infection was thought to be limited to wood rats; however, this species was isolated from the cerebrospinal fluid of two human neurobrucellosis patients in 2008 and 2011. Therefore, the non-zoonotic status of *B*. *neotomae* should be reassessed [10].

Despite more than ~500,000 new human cases being diagnosed annually, brucellosis remains under-diagnosed and neglected among livestock diseases in many endemic countries [11]. Current procedures for the microbiological isolation and typing of *Brucella* isolates are not amenable for routine diagnosis as they are expensive, time-consuming, and require the use of biohazard containment facilities. Therefore, a rapid identification method is needed to promptly identify and treat infected patients, to aid in the management of animal outbreaks, and to collect epidemiological information for surveillance systems [12].

More recently, a matrix-assisted laser desorption/ionization time-of-flight mass spectrometry (MALDI-TOF MS)-based assay has been developed for the identification of *Brucella* isolates. The assay is based on the characterization of protein profiles, and is a rapid, cost-effective, and accurate method for the analysis of biological samples [13]. Although genetic discrimination of *Brucella* species is very difficult because of their lack of variation, even minimal genomic differences among *Brucella* biovars can correlate with specific proteomic patterns. The MALDI-TOF-based assay can identify these differences, enabling genus, species, and biovar identification. Although the efficacy of the assay for typing *Brucella* strains has been evaluated [14–19], the available databases need to be improved by the addition of a greater number of strains representing the genetic variation of *Brucella*, thus increasing the discriminatory power of the databases at the species and biovar level.

In this study, we assessed a new MALDI-TOF protocol for sample preparation and constructed a database using *Brucella* strains previously characterized by specific polymerase chain reaction (PCR)-based assays that are representative of the most widespread species. To evaluate the efficacy of our method, the reference library was challenged with 197 blind-coded field samples isolated from infected animals. Finally, we evaluated the epidemiological value of the assay by comparing the clustering analysis results of 51 *B*. *melitensis* isolates obtained via the MALDI-TOF-based assay or by multiple locus variable-number tandem repeat analysis (MLVA), which is considered the most discriminatory method for *Brucella* genotyping [20–22].

## Materials and methods

### *Brucella* strains and growth conditions

All *Brucella* strains used for this study were supplied by the Istituto Zooprofilattico Sperimentale di Abruzzo and Molise (IZSAM) of Teramo (Italy), and by the Istituto Superiore di Sanità (ISS) of Rome (Italy). A total of 75 reference and field *Brucella* spp. strains, originally isolated from amongst 95 samples and identified using classical and molecular assays, were used to construct the MALDI-TOF database. These strains included: *B*. *canis* (*n* = 1), *B*. *ovis* (*n* = 1), *B*. *neotomae* (*n* = 1), *B*. *abortus* (*n* = 27), *B*. *melitensis* (*n* = 27), and *B*. *suis* (*n* = 18) (Table 1). Strains belonging to the closely related genera *Mesorhizobium*, *Ochrobactrum*, and *Rhizobium*, supplied by the Università Cattolica del Sacro Cuore (UCSC) of Rome (Italy), were also included in the database (Table 1 and S1 Table).

**Table 1:** MALDI-TOF database composition. *Brucella* strains used to construct the MALDI-TOF database. Further details regarding the *Brucella* reference strains are provided in S1 Table.

Species	Number	Biovar (N°)	Source (N°)							
			Reference strains	Human	Cattle	Buffalo	Sheep	Goat	Suine	Wild Boar
*B*. *abortus*	27	1 (8)	4	−	2	2	−	−	−	−
		3 (14)	−	−	11	1	2	−	−	−
		6 (2)	1	−	1	−	−	−	−	−
		7 (1)	1	−	−	−	−	−	−	−
		9 (2)	2	−	−	−	−	−	−	−
*B*. *melitensis*	27	1 (3)	3	−	−	−	−	−	−	−
		2 (1)	1	−	−	−	−	−	−	−
		3 (23)	1	2	1	1	14	4	−	−
*B*. *suis*	18	1 (3)	3	−	−	−	−	−	−	−
		2 (9)	5	−	−	−	−	−	2	2
		3 (2)	2	−	−	−	−	−	−	−
		4 (2)	2	−	−	−	−	−	−	−
		5 (2)	2	−	−	−	−	−	−	−
*B*. *canis*	1	−	1	−	−	−	−	−	−	−
*B*. *neotomae*	1	−	1	−	−	−	−	−	−	−
*B*. *ovis*	1	−	1	−	−	−	−	−	−	−
*Mesorhizobium spp*	1	−	1	−	−	−	−	−	−	−
*Ochrobactrum spp*	1	−	1	−	−	−	−	−	−	−
*Rhizobium spp*	1	−	1	−	−	−	−	−	−	−

*Brucella* strains were cultured on chocolate PolyViteX (PVX) agar plates (bioMérieux, Marcy-l'Étoile, France) for 48 h at 37°C in the presence of 5% CO_2_ and then inoculated into cryo-bank tubes (Mast Diagnostic, Amiens, France) for storage at −80°C until use. All other bacterial strains were cultured according to their specific growth requirements. For MALDI-TOF assays, frozen aliquots of bacteria were cultured on chocolate PVX agar plates for 48 h at 37°C in the presence of 5% CO_2_. Prior to protein extraction, the isolates were re-plated and cultured for 24 h at 37°C using the same growth conditions.

### Identification of *Brucella* strains

All *Brucella* strains used in this study were typed using specific PCR assays for genus and species identification [23], and the results of the MALDI-TOF-based identification were compared with the PCR database. Briefly, for the PCR assays, genomic DNA was extracted by heat inactivating a loop of solid bacterial culture resuspended in 200 μl of Milli-Q water. Following centrifugation, 2 μl of the supernatant were used as DNA template. The first PCR amplified a 302-bp fragment of *bcsp31*, encoding a 31-kDa cell-surface immunogenic protein in *Brucella* species, using primers *bru-cspMT* (forward; 5′-TTACCCGGAAACGATCCATA-3′) and *bru-cspMT* (reverse; 5′-AGATCGGAACGAGCGAAATA-3′) (Tarantino *et al*., unpublished). For species identification, multiplex PCR assays capable of differentiating *Brucella* species, including vaccine strains, were carried out as described previously [23].

### MALDI-TOF sample preparation

Samples were prepared as described previously [24] with some modifications. Briefly, approximately 10 colonies from PVX agar plates were suspended in 50 μl of sterile Milli-Q water and mixed carefully. Formic acid (v/v 10%) was added for bacterial inactivation and protein extraction, and 1-μl volumes of each of the inactivated reference or diagnostic samples were dropped onto eight or four spots, respectively, of a steel target plate (“non-stop” procedure). After drying the plate at room temperature, 0.5 μl of 100% ethanol was added to each well. Finally, spots were overlaid with 1 μl of reconstituted alpha-cyano-4-hydroxycinnamic acid (Bruker Daltonics, Billerica, MA). Complete inactivation of the bacteria was confirmed by plating cell lysates on PVX agar plates and incubating as previously described for 10 days.

### Stability of inactivated samples: Suitability of a “long-term” procedure for MALDI-TOF analysis

During *Brucellosis* outbreaks, samples need to be shipped to reference laboratories and thus must be prepared using a method that assures safety and protein stability. To assess the stability of the proteins and the suitability of our method for routine diagnostic testing, 98 *Brucella* strains representative of the most common species were tested by MALDI-TOF analysis immediately after the inactivation step described above (“non-stop” procedure), or after storage at 4°C for 48–72 h (“long-term” procedure). The performance of both procedures, as a percentage of correctly identified strains, was then compared.

### Identification of *Brucella* field isolates

To evaluate the efficacy of our MALDI-TOF database, 197 specimens isolated from *Brucella*-infected animals and processed by the IZSAM were investigated. Briefly, the preferred tissues from the slaughtered animals were removed aseptically and cleaned of foreign material. Small pieces were then homogenized using Stomacher bags (VWR International, Radnor, PA), seeded onto selective Farrel and Theyer-Martin-modified solid medium, and then incubated at 37°C ± 2°C in air supplemented with 5–10% (v/v) CO_2_ for up to 6 weeks. Colonies with distinctive morphology and positive urease-oxidase test were isolated and subjected to the specific PCR assays described above. After the addition of 10% formic acid for bacterial inactivation and protein extraction, as described in our protocol, extracts were stored at 4°C and then sent to UCSC within 48–72 h as blind-coded samples for MALDI-TOF investigation.

### Acquisition of mass spectra

To construct the custom reference library, the mass spectra from reference *Brucella* strains were manually acquired on a Bruker Autoflex III Smartbeam instrument (Bruker Daltonics GmbH, Bremen, Germany) in linear mode using the default parameters. Composite mass spectra were generated from eight different positions per spot using 2,000 laser shots at each spot generated by a 200-Hz Smartbeam laser (355 nm). The mass spectra were recorded at a mass/charge (m/z) range of 800 Da to 20 kDa. The instrument was routinely calibrated using an external bacterial test standard (Bruker Daltonics) prior to each mass spectra acquisition assay. To identify the blind-coded strains, mass spectra were automatically acquired using the same experimental settings.

### MALDI-TOF data analysis

Initial data analysis was performed using Bruker Daltonics MALDI Biotyper 2.0 software (Bruker Daltonics). From each selected reference strain, a main spectrum was generated from eight mass spectra according to the manufacturer’s guidelines and using default settings (Bruker Daltonics). Subsequently, four mass spectra for each of the remaining 197 blind-coded strains were acquired and compared with the generated *Brucella* library. Logarithmic score values (0–3.0) were determined by automatically calculating the proportion of matching peaks and peak intensities between the test spectrum and the reference spectra in the database. An identification was considered reliable when at least three out of four spots gave the same species identification, with a score of between 2.3 and 3.0. When the logarithmic score was < 1.7, the spectrum was reported as ‘not reliable identification’, indicating that it could not identify the genus or species of the strain. A logarithmic score of 1.7–2.299 was reported as ‘probable genus identification’, indicating that identification was reliable only at the genus level.

For the MALDI-TOF dendrogram, Main Spectrum Profiles of the previously identified *Brucella* isolates were created. Quality control of the peaks was performed using Flex Analysis software (Bruker Daltonics version 3.3) as per the manufacturer’s instructions to exclude spectra with outlier peaks or anomalies. Dendrograms were generated by similarity scoring of a set of mass spectra, and were used to represent the distance values between species determined from their reference spectra as described above [25].

### Epidemiological power of the MALDI-TOF assay

To evaluate the reliability of the MALDI-TOF assay as an epidemiological tool, we compared the clustering data obtained from 51 *B*. *melitensis* strains via MALDI-TOF analysis or MLVA. MLVA was performed using the more epidemiologically-stable MLVA-8 and MLVA-11 panels described by Le Flèche *et al*. [21] using capillary electrophoresis on an ABI-Prism 3500 Genetic Analyzer with POP-7 (Applied Biosystems, Foster City, CA) [26]. The variable-number tandem repeat fragments were sized using GeneMapper 4.1 (Applied Biosystems). We evaluated the discriminatory power using Simpson’s index, while the correspondence between the typing methods was determined using the Wallace and adjusted Wallace indexes via the online tool available from the Comparing Partitions website (http://www.comparingpartitions.info/index.php?link=Tool#) using the MLVA-8, MLVA-11, and MALDI-TOF clusters.

## Results

### Comparison of MALDI-TOF spectra of *Brucella* reference species used in the database

**Fig 1:**
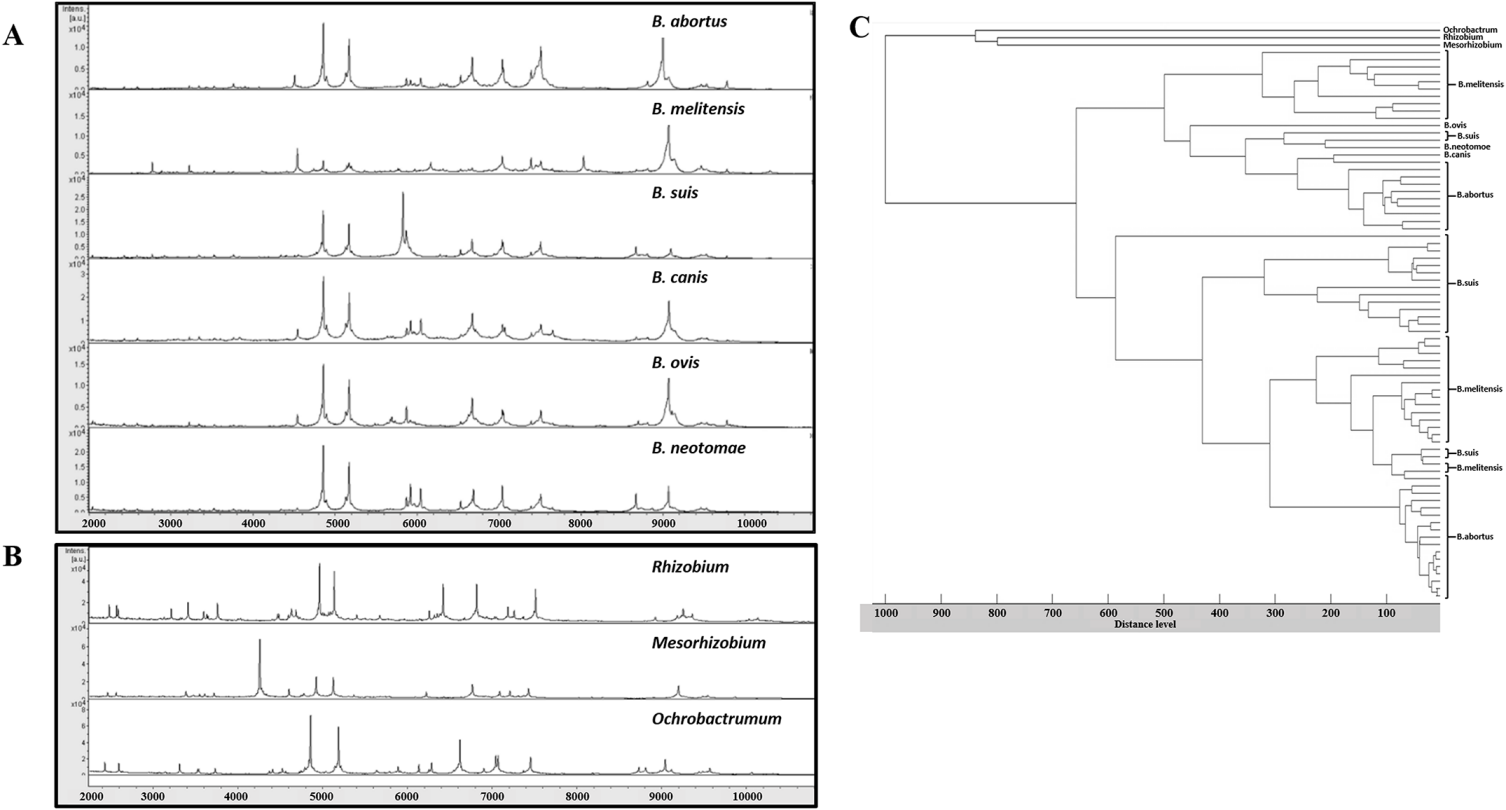
Representative MALDI-TOF protein profiles. Distinctive protein profiles of the representative *Brucella* spp. strains (A) and outgroup bacterial strains (B) included in the database. MALDI-TOF dendrogram of strains included in the database (C).

Despite the high genetic similarity of *Brucella* species, differences were observed when analyzing the representative mass spectra for each of the six *Brucella* species used for the construction of the database (Fig 1A). *B*. *abortus*, *B*. *melitensis*, and *B*. *suis*, for which a greater number of strains were examined, showed peaks with m/z within the range of 3,171.1 ± 42.3 to 12,322.5 ± 1,239.9 Da, 3,136.3 ± 64.5 to 11,503.3 ± 688.0 Da, and 3,151.9 ± 54.1 to 11,449.2 ± 449.1 Da, respectively. *B*. *canis*, *B*. *neotomae*, and *B*. *ovis*, along with the closely related bacterial species, showed peaks in the range of 3,218.4 ± 0.7 to 11,208.12 ± 7.7 Da. The *B*. *abortus* protein profile revealed four main peaks at 4,852.5 ± 1.1 Da, 7,509.6 ± 0.5 Da, 9,052.4 ± 35.2 Da, and 9,788.5 ± 1.9 Da. Similarly, *B*. *suis* showed four main peaks at 4,851.8 ± 0.5 Da, 5,828.0 ± 2.7 Da, 8,374.0 ± 2.0 Da, and 9,063.5 ± 23.0 Da. The *B*. *melitensis* profile showed only three major peaks at 7,326.8 ± 1.4 Da, 7,838.2 ± 13.5 Da, and 9,073.3 ± 2.1 Da. In addition, protein profile analysis of closely related genera *Rhizobium*, *Mesorhizobium*, and *Ochrobactrum* showed distinctive protein peaks for these species (Fig 1B), with peaks in the range of 3,094.9 ± 28.7 Da to 12,442.3 ± 1,412.6 Da. The MALDI-TOF dendrogram also highlighted the differences between the protein profiles of the *Brucella* strains and the outgroup bacteria (Fig 1C). However, the protein profiles of the *Brucella* species did not allow us to elucidate different *Brucella* clusters (Fig 1C).

### Efficiency of the MALDI-TOF database and stability of inactivated samples

The database generated in this study was evaluated using 98 previously identified *Brucella* strains using the “non-stop” protocol. As a result, 100% of samples were correctly identified at both the genus and species level (Table 2). The results of MALDI-TOF analysis of the 98 strains using the “long-term” protocol confirmed that the storage of protein extracts at 4°C for 48–72 h did not significantly affect MALDI-TOF performance. Overall, 100% and 97% of strains were correctly identified at the genus and species level, respectively, using this procedure. Of the *Brucella* strains incorrectly identified at species level, three (8.6%) *B*. *abortus* strains were identified as *B*. *suis* (Table 2).

**Table 2:** MALDI-TOF identification results. Identification of 98 *Brucella* strains tested by MALDI-TOF analysis using the “non-stop” and “long-term” protocols.

*Brucella* species (N°)	Non-stop procedure*		Long-term procedure**	
	Correct identification N° (%)	Incorrect identification N° (%)	Correct identification N° (%)	Incorrect identification N° (%)
***B*.*melitensis* (46)**	46 (100)	-	46 (100)	-
***B*.*abortus* (35)**	35 (100)	-	32 (91.4)	*B*.*suis* 3 (8.6)
***B*.*suis* (17)**	17 (100)	-	17 (100)	-
**Total (98)**	98 (100)	-	95 (97)	3 (3)

* Non-stop procedure: *Brucella* isolates were tested by MALDI-TOF immediately after the inactivation;

** Long-term procedure: *Brucella* isolates were inactivated and then stored at 4°C for 48–72 h before testing with MALDI-TOF.

### MALDI-TOF analysis of blind-coded field samples

Table 3 shows the results of MALDI-TOF analysis of the 197 diagnostic specimens collected from *Brucella*-infected animals by IZSAM and shipped, following inactivation as previously described, as blind-coded samples to UCSC. Overall, 196 out of 197 strains (99.5%) were correctly identified at the genus level, while 191 (97%) of the strains were correctly identified at the species level. In particular, all *B*. *abortus* and *B*. *suis* strains were correctly identified at the genus level (100%), while *B*. *melitensis* had an identification rate of 99.2% owing to the inability to obtain a mass spectrum for one strain. At the species level, all *B*. *suis* strains were correctly identified (100%), whereas three *B*. *abortus* strains were identified as *B*. *suis* (correct identification rate of 93.6%) and two *B*. *melitensis* strains were identified as *B*. *abortus* (correct identification rate of 97.7%). All samples were tested three times.

**Table 3:** Genus and species identification by using “long-term protocol”. Exhaustive genus and single species identification using the MALDI-TOF assay with the “long-term” protocol.

*Brucella* species (N°)	Genus identification N° (%)	No protein profile N° (%)	Species identification		
			Correct identification		Incorrect identification
			N° (%)	Average Score	Mis-identification N° (%)
***B*. *melitensis* (133)**	132 (99.2)	1 (1.1)	130 (97.7)	2.496	2 (2.3)^a^
***B*. *abortus* (47)**	47 (100)	−	44 (93.6)	2.468	3 (7.4)^b^
***B*. *suis* (17)**	17 (100)	−	17 (100)	2.710	-
**Total (197)**	196 (99.5)	1 (0.5)	191 (97.0)	-	5 (2.1)

^a^Mis-identified like *B*. *abortus*;

^b^Mis-identified like *B*. *suis*

### Effectiveness of MALDI-TOF as an epidemiological tool

**Fig 2:**
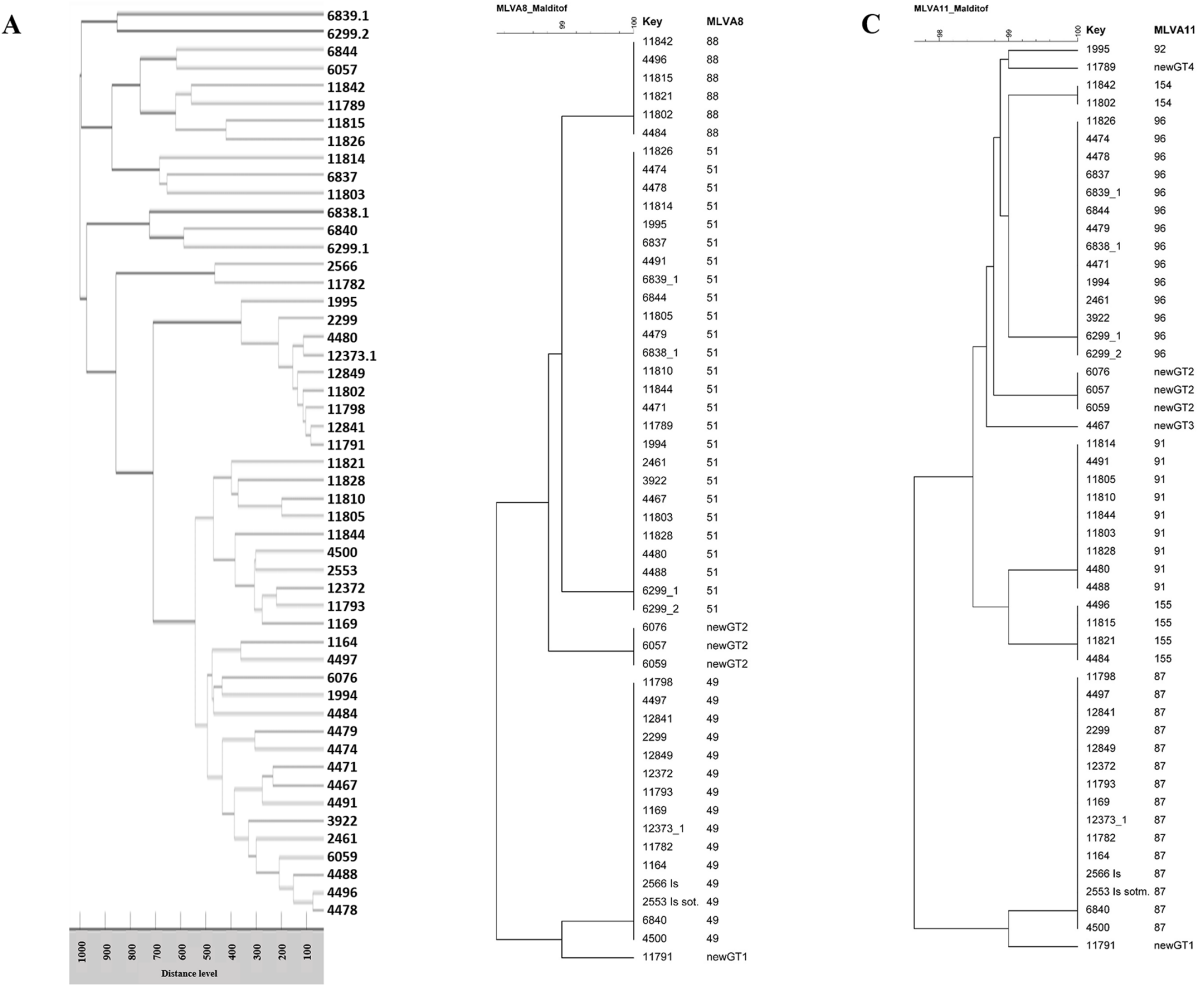
Representative dendrograms of the MALDI-TOF, MLVA-8 and MLVA-11 assays. MALDI-TOF dendrogram of 51 selected *B*. *melitensis* field strains from Italy (A). Unweighted pair group method with arithmetic mean assessments of the relationships between the 51 *B*. *melitensis* isolates using MLVA-8 (B) and MLVA-11 (C) data. Each strain is individuated by an identification number and genotype. Genotype designations followed the nomenclature used in the international MLVA database (http://mlva.u-psud.fr/mlvav4/genotyping/).

To evaluate the reliability of our MALDI-TOF assay as an epidemiological tool for *Brucella* genotyping, clustering data obtained from MALDI-TOF or MLVA analysis of 51 *B*. *melitensis* field isolates were compared. As shown in Fig 2A, the resulting MALDI-TOF dendrogram, obtained from similarity scoring of the mass spectra acquired for identification, highlighted seven major clusters at a distance level of 700. Conversely, the MLVA-8 scheme identified five genotypes (Fig 2B), while the MLVA-11 scheme identified 10 genotypes (Fig 2C).

The discriminatory power of the MALDI-TOF clusters and MLVA was then assessed by evaluating Simpson’s index of diversity. Table 4 shows the diversity coefficients for the MLVA-8 and MLVA-11 panels and the MALDI-TOF partition according to the clustering, and reveals comparable resolution for the MALDI-TOF and MLVA-8 methods. The Wallace coefficient for comparing the congruence between type assignments showed a strong correlation between MLVA-8 and MLVA-11, but poor correlation was found for MALDI-TOF against the MLVA-8 and MLVA-11 schemes (Table 5). The Wallace index for MALDI-TOF against MLVA-8 and MLVA-11, although fairly good, had the expected Wallace under independence included in the calculated 95% confidence intervals for the Wallace index. In these cases, a large part of the measured agreement was due to chance because the adjusted Wallace index was very close to zero (Table 5). In all other comparisons (MLVA-8 vs. MLVA-11 and reverse), the Wallace index was significantly higher than the expected value under independence, demonstrating that the congruence between the MLVA-8 and MLVA-11 typing methods could not be attributed to chance alone.

**Table 4:** Simpson’s diversity indices. Simpson’s diversity index and respective 95% confidence intervals with partitions for MALDI-TOF-MS clusters and MLVA-8 and MLVA-11 panels.

Method	Partitions (n°)	Simpson’s ID	CI (95%)
MALDI-TOF	10	0.707	(0.589–0.826)
MLVA-11	10	0.810	(0.750–0.870)
MLVA-8	5	0.649	(0.553–0.744)

**Table 5:** Wallace values. Wallace under independence, Wallace, and adjusted Wallace index values and respective 95% confidence intervals for MALDI-TOF-MS clusters and MLVA-8 and MLVA-11 panels.

Method A	Method B	*W*_i(A→B)_	*W*_A→B_ (95% CI)	*AW*_A→B_ (95% CI)
MLVA-11	MLVA-8	0.351	1.000 (1.000–1.000)	1.000 (1.000–1.000)
MLVA-8	MLVA-11	0.190	0.540 (0.441–0.639)	0.432 (0.310–0.555)
MALDI-TOF	MLVA-8	0.351	0.357 (0.225–0.488)	0.008 (0.000–0.211)
MLVA-8	MALDI-TOF	0.293	0.297 (0.155–0.438)	0.006 (0.000–0.206)
MALDI-TOF	MLVA-11	0.190	0.196 (0.125–0.267)	0.007 (0.000–0.095)
MLVA-11	MALDI-TOF	0.293	0.302 (0.176–0.427)	0.013 (0.000–0.190)

Bold values: the confidence interval of *W*_*A*→*B*_ includes the Wallace in case of independence (Wi).

## Discussion

MALDI-TOF MS is a rapid, accurate, and cost-effective method for microbial characterization and identification. Its strength can be attributed to the characteristic and unique protein profiles that are generated for each microorganism, which provide an accurate microbial identification at the genus and species levels. Because of these favorable characteristics, MALDI-TOF-based assays are replacing/complementing conventional techniques for routine identification of microorganisms in clinical microbiology laboratories [13,27,28]. The resolution and accuracy of MALDI-TOF MS allow precise identification of most Gram-positive and Gram-negative bacterial species, making it a reliable approach to identify highly pathogenic organisms (e.g., *Brucella* spp., *Coxiella burnetii*, *Bacillus anthracis*, *Francisella tularensis*, and *Yersinia pestis*), including those that could be used as agents of bioterrorism [15,29,30]. Conventionally, these organisms are identified using phenotypic, genotypic, and immunological tests that are slow, cumbersome, and exhibit significant risk to laboratory personnel. However, in the case of biological warfare or when natural outbreaks occur, timely detection and identification of the causative agent is essential for developing a prompt and effective response.

*Brucella* species are characterized by extremely high levels of nucleotide similarity, although they vary widely with regards to host tropism, microbial and disease phenotypes, and pathogenicity. The development of molecular typing tools was hampered by this lack of diversity for many years. In addition, although the MALDI Biotyper 2.0 Standard Database (Bruker Daltonics) is used for routine identification of microorganisms in clinical microbiology and contains > 3,000 specific mass spectra from various bacterial and fungal species, spectra for members of the genus *Brucella* are not present in the database. This severely limits the application of this approach in high incidence countries where *Brucella* species are frequently isolated from patients. Numerous groups have tried to optimize *Brucella* identification using a supplemented library containing *Brucella* species [14,16], or by using a custom *Brucella* library [15]. Although these supplemented databases allow accurate identification, the strains used to challenge the database are often the same as those used for its construction. Moreover, the protein extraction methods are time consuming and often complex and increase the infection risk for the operator.

In this study, we assessed a modified protein extraction protocol for MALDI-TOF analysis to improve the identification accuracy, minimize sample manipulation, and to make the method more suitable for routine diagnostic testing. We constructed a custom database using previously characterized human and animal *Brucella* strains that were representative of the most widespread species, but focusing on *B*. *abortus*, *B*. *melitensis*, and *B*. *suis*. Because of their genetic similarity, strains belonging to the genera *Rhizobium*, *Mesorhizobium*, and *Ochrobactrum* were also included [31].

Unlike previous studies [14], which observed characteristic peak profiles at the genus level but not at the species level, the reference spectra generated using our extraction protocol revealed different peptide mass fingerprints for the six representative *Brucella* species, showing specific peaks for each species. All reference *Brucella* strains used for the construction of the database were correctly identified when tested as blind-coded samples, except *B*. *canis*, *B*. *ovis*, and *B*. *neotomae*, which were correctly recognized at the genus level but not at the species level. However, this was likely the result of the limited number of these species included in the database (data not shown). In addition, MALDI-TOF analysis of closely related genera showed distinctive spectra compared with the *Brucella* species, and none of bacterial samples belonging to other genera were identified as *Brucella* (data not shown). These results suggest that our extraction method and database can be used to accurately identify *Brucella* at the species level, but that an increased number of reference strains would improve the identification accuracy of the less represented species.

Interestingly, our procedure does not affect MALDI-TOF performance. Overall, 100% and 97% of strains were correctly identified using the “non-stop” and “long-term” protocols, respectively, allowing shipment of the samples to the reference laboratories without the biological safety risk to the laboratory personnel. This approach could be advantageous for rapid identification of outbreak strains and could reduce the cost of identification. To confirm the suitability of this approach as a standard laboratory testing procedure, 197 samples, isolated from *Brucella*-infected animals by IZSAM, were prepared according to the “long-term” procedure and sent to UCSC for MALDI-TOF analysis. As with the preliminary tests, 99.5% and 97% of field samples were correctly identified at the genus and species level, respectively. Only one *B*. *melitensis* isolate failed to generate a spectrum, probably because of an insufficient quantity of inoculum during protein extraction. However, the two *B*. *abortus* isolates misidentified as *B*. *suis* should be investigated further using both genomic and proteomic approaches, even though the dendrogram generated using *Brucella* strains contained in the database highlighted the close genetic relationship between *B*. *suis* and *B*. *abortus*. Interestingly, despite the small number of samples examined in the current study, we observed that all *B*. *suis* isolates were correctly identified at the species level (100%) in each experiment. This may be because *B*. *suis* is the most genetically divergent and well-characterized species within the genus *Brucella* [32].

Although culture conditions might significantly affect microbial protein expression (Welker et al., 2011), our results generated using field specimens confirmed previous reports indicating that MALDI-TOF-based identification is not influenced by different culture conditions, culture formulations, or cultivation time [14,28,33]. Further, the modified extraction protocol developed in the current study was faster than the Bruker method while still ensuring high laboratory safety and identification rates. The inactivation procedure is in accordance with guidelines for the complete inactivation of highly pathogenic bacteria, i.e. biosafety level 3 agents, including *Brucella*.

Recent studies have suggested that MALDI-TOF-based analysis methods can be used as an epidemiological tool for surveillance purposes [34]. Therefore, to evaluate the epidemiological value of our method, we tested 51 *B*. *melitensis* isolates and compared the MALDI-TOF clustering analysis results with those obtained via MLVA, which is considered the gold standard testing method. MLVA is one of the most suitable tests for detecting genetic variance within genera with high genomic identity, such as *Brucella*. Currently, this method is widely used for epidemiological monitoring of brucellosis and for tracking the source(s) of infection [20–22,35]. The MLVA clustering of *B*. *melitensis* from Italy using the 8- and 11-locus panels allowed us to elucidate the true phylogenetic relationships within the Italian population. All of the Italian strains tested were isolated from diseased livestock and slaughtered according to the eradication plan. The strains were divided into five genotypes based on the MLVA-8 analysis and 10 genotypes using MLVA-11, and were similar to the most prevalent Italian genotypes reported by Garofolo et al. [35]. The strains used in the comparison test were truly representative of the Italian *B*. *melitensis* population and thus formed the right test population to evaluate the epidemiological resolution of the MALDI-TOF analysis method. MLVA is a useful tool for tracing disease origins, monitoring the spread of disease, studying population dynamics, and even for discerning endemic from epidemic patterns [36,37]. The MALDI-TOF technique used in the current study could not reproduce the MLVA clustering, highlighting its inability to decipher the true phylogenetic tree. While the diversity index scores were good, confirming a relatively high discriminatory power, the correlation with the two MLVA panels was fairly poor, suggesting that the MALDI-TOF protein profiles do not correspond with the genetic evolution of *Brucella*. These results again showed that MALDI-TOF identification, based on the bacterial proteome, is affected by minor genetic differences that do not appear in MLVA analyses [15]. However, it would be interesting to examine whether proteomic differences are related to a diverse pathogenesis. In this case, proteomic (MALDI-TOF) and genomic (MLVA) approaches could be used in tandem for a more complementary and in-depth analysis.

Based on our preliminary results, the MALDI-TOF-based method described here is fast and highly reliable for routine identification and discrimination of *Brucella* isolates at the genus level, thus providing an actionable result with regards to laboratory safety and public health. Moreover, it has very good positive identification rates for *B*. *melitensis*, *B*. *suis*, and *B*. *abortus*. However, the library should be further supplemented to increase the accuracy of MALDI-TOF identification at the species and subspecies level and to achieve a more efficient tool for epidemiological studies, thus replacing the current molecular identification techniques.

## Supporting information

S1 Table Reference strains used in this study. (TIF)
