# Supplementary figures and images for: Rapid and safe one-step extraction method for the identification of Brucella strains at genus and species level by MALDI-TOF mass spectrometry
Source: PLoS One. 2018 Jun 5;13(6):e0197864. doi: 10.1371/journal.pone.0197864 (PMC5988274; doi:10.1371/journal.pone.0197864)

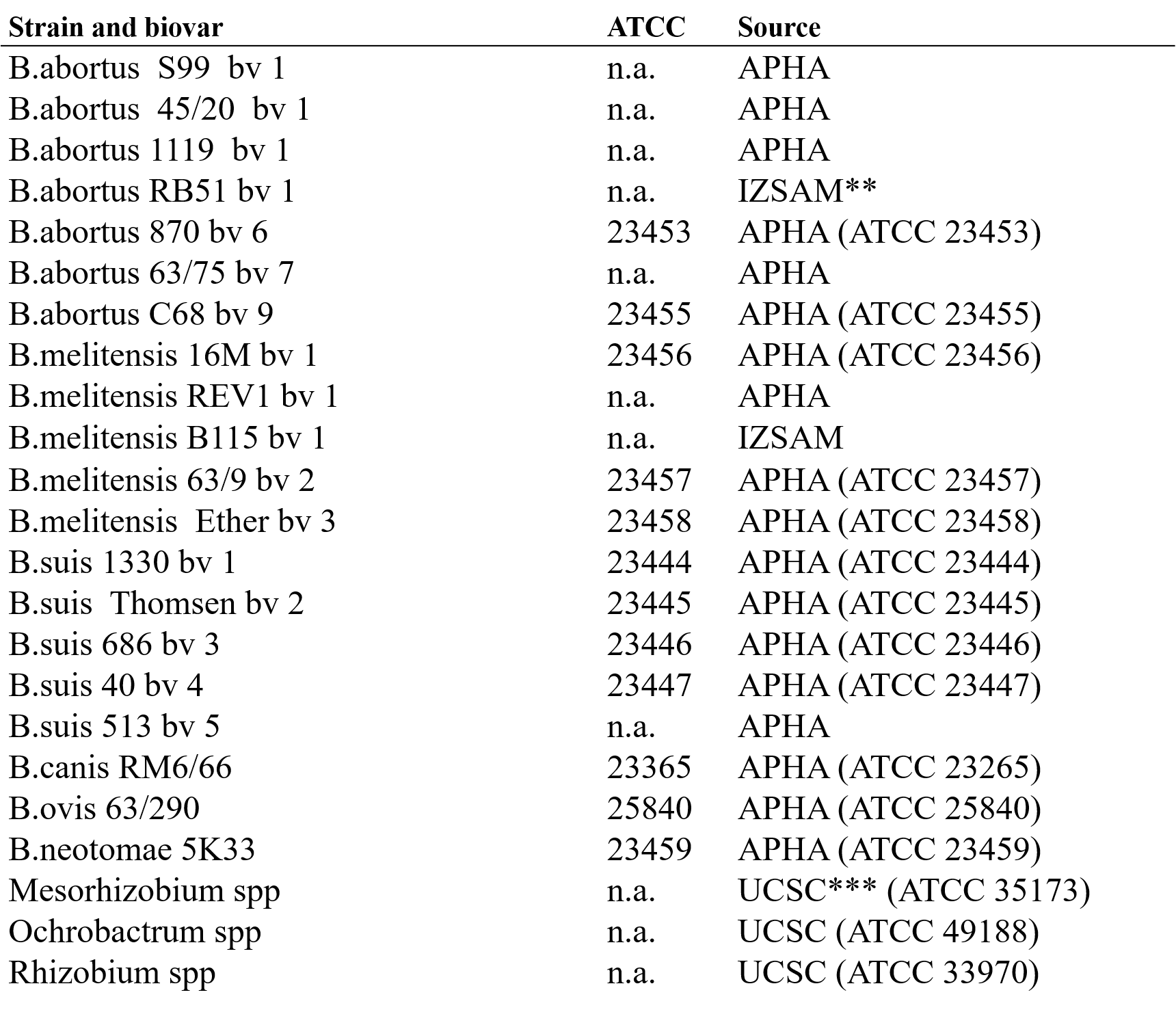

Supplement: S1 Table — (TIF) [file pone.0197864.s001.tif]
